# The Pathology of, and the Treatment of Ulcers, Varicose Veins and Cutaneous Diseases of the Leg by Martin’s Pure Rubber Bandage

**Published:** 1879-11

**Authors:** F. H. Stuart


### Selections.

---

### THE PATHOLOGY OF, AND THE TREATMENT OF ULCERS, VARICOSE VEINS AND CUTANEOUS DISEASES OF THE LEG BY MARTIN'S PURE RUBBER BANDAGE.

By F. H. STUART, M.D.

The lower extremity is the seat of disease of various forms. Its dependent position and its constant exposure to injuries serve as the exciting causes of extensive and protracted disease. The most common and important of those diseases are ulcer, eczema and varicose veins. They are painful, all of them. They undermine the health, even when the health is not already broken, and the individual thus predisposed to such disease. Indeed, it would not be difficult to maintain the position that broken health is a necessary antecedent of most of the diseases referred to. At any rate, in most instances, when these cases come under the care of the surgeon the disease has existed for a long time. Many domestic remedies have been tried, all with a like discouraging result. At last a doctor is seen; this ointment or that is applied, his blood (which is "out of order" and has caused the trouble) is prescribed for, but still there is no relief, or, at best, it is only temporary. Another doctor is consulted, who, at the second visit, is probably told that that "stuff" (meaning ointment, or medicine, or both) is the same Doctor A. gave, and it did no good. So the story might be continued. In short, these diseases of the lower extremity have been a bugbear to physician and surgeon—the latter having little better success in treating them than the former.

The number of cases is great, and a successful method of cure will confer the blessing of comfort, health and vigor to many. But a successful and permanent cure will depend upon a correct appreciation of the causes of these diseases and the relation of these causes to the conditions found. Pre-eminently, *treatment must be based upon pathology.* Professor S. D. Gross says: "It may confidently be asserted that there is not, in the whole domain of surgery, a class of maladies, the *pathology* and *treatment* of which are less understood by the profession generally, than those of ulcers."

It is difficult to formulate the pathology of ulcers. Yet the phenomena attending their formation can be so described as not to raise disputed questions or provoke discussion of the meaning of terms.

An ulcer is the last stage of a pathological process. This pathological process we must study, if we would successfully and truly cure the ulcer. The chief elements of this process are (1) deranged circulation, and (2) impaired or arrested nutrition. These two involve a third (3), disturbed innervation. Each of these react upon and aggravate the others. These are the three factors in all ulcerations. It concerns us now only to study them in relation to ulcers of the leg.

I.—*Deranged Circulation in the Leg.*—This is brought about by a variety of causes. Among these I mention: much standing without change of posture, constriction of limbs by garters, exertion, and any other way in which the onward current of the blood is impeded or obstructed. The result is a gradual yielding of the coats of the vein. This distension of the veins retards the flow of blood. The capillaries in turn enlarge in diameter, and permit exudation of serum and even of cells.

The valves of the veins are separated, leaving a chink between them. They soon atrophy, becoming in time entirely useless. An effort is sometimes made to compensate for the dilatation by a thickening of the walls of the vein by connective tissue between the muscle cells. They may also be thickened by plastic infiltration and organization in the outer or cellular coat.

It is commonly thought that the deep veins are not subject to the varicose condition, being compressed and supported by the muscles. But this idea is not correct The circulation in these is frequently interrupted by the contraction and pressure of the muscles upon them. Provision for this is made in that the valves are more numerous in the deep than in the superficial veins. It is to be remembered that the valves do not act at all in aiding the onward progress of the blood, but by supporting the column of blood above them. They are only closed when the current is interrupted, when they serve to distribute the blood tension in the portion of the vein below them.

This condition of weakness of veins and disturbed circulation usually only obtains in persons whose general health is below the normal, so that not only is the blood supply disturbed, but the blood quality defective.

II.—*Impaired or Arrested Nutrition in the Leg*.—This, from one point of view, is but a corollary to what has just been said. The integrity of the tissues depends first of all upon the blood supply being regular and sufficient. This cannot be in the condition just spoken of. But ulcers often exist independently of varicose veins, or any other *visible* disturbance of the circulation. "A state of nutritive equilibrium" is dependent not alone upon the blood supply. The conditions upon which it depends are both local and general. From some cause or causes, as exposure to heat or cold, pressure, injury, and many other agents, the tissues may imperfectly or entirely fail to appropriate the elements from the blood necessary to their growth and maintenance. Or the nutrition may be affected through the blood by the quantity and quality of the food, by changes in the blood itself, by exercise, by occupation, by climate—in short, by all the conditions, internal and external, which affect and determine the general health. The nutritive process may, in a sense, be still going on, but the products are abnormal.

I have hitherto purposely avoided the use of the word *inflammation.* I have preferred to refer to some of the phenomena observed in the course of these diseases of the leg. These phenomena, and others to which I shall refer, are the phenomena which, *collectively*, are usually present in the condition called "inflammation." The term is indefinite. It describes nothing. It means more or less, according as we observe, analyze and study the abnormal process. "Their effect," as Carpenter says, "is to produce a depression in the vital powers, which manifests itself in *a deficient as well as abnormally directed formative activity*, and an *increased tendency to degeneration.*"

III.—*Disturbed Innervation.*—The influence of the nervous system in maintaining the integrity of the tissues and the function of the different parts of the body is clearly established. *How* it does so, is yet a matter of debate. In the diseases under consideration there is intimate connection between the cerebro-spinal nervous system and the sympathetic system. Both sets of nerves are disturbed. The vaso-motor nerves by the dilatation of the capillaries become exhausted. Morbid nutrition is caused by nerve disturbances as well as by nerve defect.

The pain of these diseases is sometimes excessive. This is often due to pressure upon the nerve by the distended veins, for the pain is greatest when the patient is standing or sitting; "but if the leg is lifted up, and the blood vessels so emptied, the pain is quickly relieved." Or pain may be caused by destruction of nerve tissue, or by stretching and compression. It is also to be remarked that the irritation of the pain affects the general health, as well as the local condition.

The phenomena thus briefly described usually co-exist in the diseases of the leg referred to in this paper. It is seldom that one is found alone. We *may* have varicose veins without much appreciable disease besides; but exudation and infiltration eventually follows. The part comes to be in the state called chronic inflammation. "This inflammation may be located in the depths of the cutis, in the cellular tissue, muscles, glands, periosteum or bones. * * * * * The skin is traversed by dilated vessels, hence it is redder than normal; it is swollen, partly from serous, partly from plastic infiltration, and is sensitive to pressure. * * * * * The papillæ are larger and more succulent; the development of the cells of the rete Malpighii also become more plentiful; its superficial layers do not pass into the normal horny state; the connective tissue of the papillary layer is softer and becomes partly gelatinous."

The ulcer results from a bruise or other injury to *tissues that are already in a state of disease-lowered vitality.* But these injuries cannot be said to be other than the exciting causes. Cuts and bruises are every day made upon healthy skin. These heal readily, and leave only a slight cicatrix, or no cicatrix at all. It is not easy to determine the cause of this condition of lowered vitality. Most likely it is not a local cause. But the leg, being removed from the centre of circulation, becomes the seat of manifestation of the *dyscrasia.* The ulcer may be healed, cicatrization may be brought about, but there remains the chronic inflammation of the surrounding tissues. The skin remains bluish or brownish red—a condition of lowered vitality that makes it ready to break down in ulceration again as soon as the limb is used. (See case No. 1.)

I need not dwell in particular upon the pathology of eczema of the lower extremity, after what has already been said.

In regard to diagnosis of ulcers of the leg, it is to be observed that varicose ulcers are situated below the middle of the leg, and usually are single. They are to be distinguished from syphilitic ulcers, which are multiple, and exist usually above the middle of the leg. Mr. Hilton gives this explanation of why these ulcers so frequently occur at the inner and lower part of the leg: "The superficial and deep veins of the leg freely communicate with each other in the neighborhood of the ankle-joint. The first two inches above that point is the spot where the greatest stress is laid upon these superficial veins; below that point they freely communicate, and if the blood cannot return by the superficial veins, it can do so by the deep veins, and *vice versa.*"

To enter upon the description and classification of ulcers would too much extend this paper. My object has been merely to call to mind general principles—a broad basis, which gives the best vantage-ground for practice.

Nor can I refer to the almost countless and frequently worthless means of treatment which have, from time to time, been proposed. The characters of ulcers are so various and usually so well marked, that the appropriate treatment can only be selected after a careful diagnosis. Each case claims a particular study. But just as there are general principles of pathology underlying their formation, so there are general principles of treatment applicable to a great variety of cases.

The first step in the process of healing seems to be the subsiding of the swelling in the surrounding tissues. Hence whatever tends to remove this swelling promotes the healing process. One of the most important and certain means of securing this is by compression, regularly applied. Bandages of various kinds have been used for this purpose for many years. Plaster dressing and adhesive plaster have also been recommended. The success of Baynton's method is due to the compression of the vessels and tissues, and not, as he supposed, to the effect upon the lymphatics alone. This is one of those curious instances where a practical man suggests a valuable remedy, but gives an erroneous explanation of its action.

In the pure rubber bandage of Dr. Martin, of Boston, is found a most valuable addition to the means of cure of a large class of diseases. It meets indications that are almost essential to success. My experience of over a year does not not warrant me in speaking of it as enthusiastically as its author does. Yet cases of ulcer, varicose veins and eczema have improved and been cured with *remarkable* rapidity; and *in every case* the expression of *comfort* from its use has been very emphatic. "I would not be without it," "I would not take five dollars for it," "I would not part with it"—are common forms of expressions after a patient has used it for a short time. Although ulcers, varicose veins and eczema may be cured by other means, the great advantage of the rubber bandage is, that it permits patients to go about and pursue their avocation. They do this with great comfort, and without at all interfering with the process of cure. Indeed, I am of the opinion that it is a great aid to cure for patients to be about. The general health is invigorated, and they experience the tonic effect of feeling and being as comfortable and vigorous as ever. Every one has observed how curative this mental state is in almost every class of disease. It is a therapeutic measure eagerly to be sought after.

It may be well to inquire how the pure rubber bandage accomplishes the result in these cases. It does so by the gentle, equal and continuous pressure it maintains. It supports and assists the capillary circulation. This pressure also promotes the absorption of the fibrinous or serous deposits in the tissues. The rapidity of the absorption is sometimes very surprising. (See case No. 2.) It is not necessary to raise the question as to how this is accomplished—whether by the effect upon the lymphatics or by the capillaries. It also mechanically expels the blood from the over-distended and weakened veins, which it supports and compresses. Often there is a weakened condition of the heart. In these cases the feeble circulation is aided by the elasticity of the bandage. The continuous warmth and moisture, and the exclusion of the air, are other elements contributing to the favorable result.

The mode of application is important. It should be applied so as to make *gentle, even pressure.* It should not give the sensation of squeezing. It is only necessary to put it on tight enough to keep its place. It does not readily slip. Each fold should overlap the previous one about a half an inch. It is not necessary to make any reverse turns—only to wrap it round continuously. Its elasticity makes it fit everywhere with equal smoothness and comfort. It should always be begun at the toes.

It is applied directly to the surface, no protective being anywhere necessary. I always direct the patient to take it off after getting into bed, having previously made ready two basins of water by the bed—one to sponge the leg and the other to wash off the bandage. Then simply cover the ulcer or eczematous patch with muslin, so as not to soil the bed-clothing. The bandage is hung over a chair till morning. It is to be re-applied *before rising.* No ointment or grease of any kind should ever be used, as it soon destroys the rubber. * * * * *
—*Proceedings of the County of Kings Society.*

